# Primary health care networks and impacts in low- and middle-income countries: a systematic review

**DOI:** 10.1093/heapol/czag003

**Published:** 2026-01-16

**Authors:** Dominic Dormenyo Gadeka, Genevieve Cecilia Aryeetey, Helen Bour, Henry Okudzeto, Patrick Addo, Noemia Teixeira de Siqueira Filha, Bassey Ebenso, Helen Elsey, Irene A Agyepong

**Affiliations:** Department of Health Policy, Planning and Management, University of Ghana School of Public Health, P.O. Box LG13, Legon, Accra, Ghana; Department of Health Policy, Planning and Management, University of Ghana School of Public Health, P.O. Box LG13, Legon, Accra, Ghana; Department of Health Policy, Planning and Management, University of Ghana School of Public Health, P.O. Box LG13, Legon, Accra, Ghana; Department of Health Policy, Planning and Management, University of Ghana School of Public Health, P.O. Box LG13, Legon, Accra, Ghana; Department of Health Policy, Planning and Management, University of Ghana School of Public Health, P.O. Box LG13, Legon, Accra, Ghana; Department of Health Sciences, University of York, York, YO10 5DD, United Kingdom; Leeds Institute of Health Sciences, University of Leeds, Leeds, LS2 9NL, United Kingdom; Hull and York Medical School, University of York, York, YO10 5DD, United Kingdom; Faculty of Public Health, Ghana College of Physicians and Surgeons, P.O. Box MB 429, Accra, Ghana

**Keywords:** primary health care, health provider network, low- and middle-income countries, health outcomes, health service delivery, networks of care

## Abstract

Primary healthcare provider networks (PHCPNs) are increasingly recognized as promising strategies to effectively strengthen health systems in low- and middle-income countries (LMICs). However, there is limited information on the influence PHCPNs may have on the process and clinical outcomes of health services. This study sought to answer the questions: what is the extent, range, and nature of research on PHCPNs in LMICs, what are the types of PHCPNs described, and what are the processes, e.g. access to care, coverage of health services, quality of care and services, safety of care, and the clinical care outcomes of PHCPNs reported in the published literature? We report on a systematic mixed-methods review on PHCPNs as a strategy to strengthen health systems in LMICs following the PRISMA guidelines. The quality of the included studies was assessed using the ROBINS-I and Mixed Methods Appraisal tools, while a narrative synthesis was employed to describe the results. Fifteen primary studies were found eligible for the review. From the included papers, eight types of PHCPNs were identified across various contexts and countries. We found that the PHCPNs primarily focus on maternal, newborn, and child health outcomes. The study reveals that: (i) PHCPNs contribute to improvements in the process outcomes of health services by enhancing access to care, coverage of health services, quality of care and services, and safety of care, and (ii) they support improvements in clinical outcomes by helping to reduce maternal, neonatal, and perinatal mortalities and stillbirths. This body of literature we reviewed suggests that PHCPNs make a difference in the process and clinical outcomes of health services in LMICs. This review serves as both a mapping and clarification exercise to promote the adoption of PHCPNs and as a foundation for further research, especially in areas of health services beyond maternal, newborn, and child health.

Key messages

- This paper contributes to the literature on primary healthcare provider networks (PHCPNs) and their role in implementing primary healthcare (PHC).
- PHCPNs make a difference in the process and clinical outcomes of health services by improving access to care, expanding health service coverage, enhancing the quality and safety of care, and supporting better clinical outcomes by reducing maternal, neonatal, and perinatal mortalities and stillbirths.
- The impacts of PHCPNs are across various types of PHCPNs (public, private, or mixed public-private facility ownership) and in diverse country contexts, including both rural and urban areas.
- There is a need for continued work on designing, implementing, and evaluating provider networks in PHC in low- and middle-income countries, especially in areas of health services beyond maternal, newborn, and child health.

## Background

Resilient and equitable health systems require robust primary health care (PHC) (Van Weel and Kidd 2018). PHC is the first point of contact between patients/clients and the healthcare system, and it is recognized as a pathway to universal health coverage (UHC) (World Health Organization 2018). A well-developed PHC system ensures long-term effective relationships with patients while providing health promotion, disease prevention, treatment, rehabilitation, and palliative care services. However, while evidence abounds that PHC improves population health and healthcare systems’ effectiveness, responsiveness, and efficiency, it remains the weakest component in many countries’ health systems, particularly low and middle-income countries (LMICs), despite systematic efforts to strengthen it (Bitton et al. 2019, Qian and Ramesh 2024). Significant bottlenecks include poor referral systems and fragmented service delivery, inefficient provider-payment mechanisms, limited coverage of services, and inadequate capacity to deliver the basic package of PHC (Primary Health Care Performance Initiative-phcpi; Results for Development-R4D; Joint Learning Network-JIN 2023, Ghana Health Service 2024).

Primary healthcare provider networks (PHCPNs) are increasingly recognized as promising strategies to address these gaps and effectively strengthen health systems in LMICs (Bhatta et al. 2020, Cordier et al. 2020, Fasawe et al. 2020, Martinez Vergara et al. 2020, Kalaris et al. 2022, Kamanga et al. 2022). PHCPNs are “a group of public and/or private health service delivery sites deliberately interconnected through an administrative and clinical management model, which promotes a structure and culture that prioritize client-centered, effective, efficient operation and collaborative learning, enabling providers across all levels of care and the community to work in teams and share responsibility for health outcomes” (Carmone et al. 2020). The use of PHCPNs for strengthening health systems is supported by implementation science theory, which emphasizes the importance of networks in policy implementation (Hanf et al. 1978, Hjern and Porter 1981). For instance, bottom-up theorists, Hanf and colleagues, specifically demonstrated that policy implementation through networks, such as networks of frontline service providers, is more successful than implementation carried out through a top-down approach (Hanf et al. 1978).

Since the adoption of PHCPNs by some LMICs, empirical evidence attributes significant improvement in the delivery of PHC services to the networks, by reducing fragmentation in coordinating referrals and services, ensuring accessible PHC for diverse communities, and leveraging limited funds and human resources to maximize performance at all levels of the healthcare system (Ghana Health Service 2024, Results for Development 2024). Also, there is evidence of increased collaboration and mutual technical and operational support among facilities, and exchange of knowledge and information (Systems for Health Ghana 2019, Debpuur et al. 2021,Debpuur C, 2021, Fenny AP, Chikhradze T, Hammah E, Dzakpasu A. 2021 2023). Moreover, an earlier review of network literature in LMICs indicated that provider networks enhance PHC at multiple levels, including the patient/household or community and the health facility levels (Gadeka et al. 2023).

While PHCPNs offer numerous potential advantages, their adoption remains uneven and is marked by significant challenges, including limited understanding regarding their impacts on process outcomes, such as access to care, service coverage, quality of care and services, and safety of care, which could assist policymakers in incorporating them into national health strategies (Carmone et al. 2020, World Health Organization 2024). Furthermore, evidence regarding the influence of networks on clinical outcomes in LMICs, including mortality rates, is sparse (Carmone et al. 2020, World Health Organization 2024). Recent scoping review studies that mapped the landscape of PHCPNs in LMICs specifically underscore the need to improve the efficiency, quality, and reach of health services in resource-limited environments through PHCPNs (Carmone et al. 2020, Agyemang-Benneh et al. 2023, Kalaris et al. 2023). Building on these scoping reviews by gaining insights into the impact of PHCPNs on both process and clinical outcomes of health services in LMICs could foster their adoption, enhance process outcomes, and improve overall health outcomes. Understanding how networks can be implemented in complex environments, such as rapidly growing urban areas with a plurality of providers, is a further urgent priority.

This article presents findings from a systematic review of the literature on PHCPN and their impacts on service delivery processes, e.g. access to care, coverage of health services, quality of care and services, safety of care, and outcomes in LMICs to contribute to policy and program design and implementation of PHCPN in LMIC.

## Methods

We followed the Preferred Reporting Items for Systematic Reviews and Meta-Analysis (PRISMA) criteria (Supplementary file S1) to guide the conduct and reporting of the review (Page et al. 2021). We chose a systematic rather than a scoping review as this study is preceded by a scoping review, which was undertaken to understand how primary care networks in low-income and lower middle-income countries (LLMICs) are conceptualized, implemented, and analysed in the literature and further explored the evidence of the effectiveness of these networks (Agyemang-Benneh et al. 2023). This study aims to gain insights into the impact of PHCPNs on both process and clinical outcomes of health services in LMICs, which was not considered in the previous study, but which could foster the adoption of PHCPNs, enhance process outcomes, and improve overall health outcomes. The study was registered with the Open Science Framework: http://osf.io/ptu9c.

### Identifying the research question

Our research question was ‘What is the extent, range and nature of research on PHCPNs in LMICs, and what is the evidence from this literature on the impact of PHCPNs on process and clinical outcomes of primary health care services in LMICs?’

### Eligibility criteria

For eligibility criteria, we were guided by the definition of PHCPNs (Carmone et al. 2020). Using the definition and focusing on the LMIC context, we defined the following inclusion and exclusion criteria for the systematic review (Table 1). We excluded studies on primary care networks in high-income countries because our goal is to explore the literature to provide an in-depth LMIC context-specific understanding of the impact of PHCPNs in terms of process and clinical outcomes to contribute to policy and program design and implementation of PHCPNs in LMIC.

**Table 1:** Inclusion and exclusion criteria.

Inclusion criteria	Exclusion criteria
Networks that operate at the level of primary healthcare or that extend from home/community to primary through to tertiary levels	Networks that operate solely at the secondary and/or tertiary levels of the healthcare system
PHCPNs in LMICs as defined by the World Bank (The World Bank 2024)	PHCPNs in high-income countries as defined by the World Bank (The World Bank 2024)
Primary research studies, including descriptive case studies on PHCPNs in LMICs, published in English	Literature reviews, technical reports, letters to editors, unpublished studies and documents/reports that discussed PHCPNs that had not been implemented, published in English and any other language

### Identifying relevant studies

A broad electronic search strategy was employed to identify all relevant primary research studies conducted in LMICs and reported in the English language, with no date restrictions. To ensure the quality of the findings, we excluded unpublished studies. Additionally, no date limits were applied to enable the collection of all relevant studies to ensure the richness of the data to answer the research question. We initially searched PubMed, Embase (Ovid), SCOPUS, and PubMed Central databases in October 2024 using keywords including “primary healthcare provider networks,” “primary care networks,” “networks of practice,” “networks of care,” “care networks,” “health facility networks,” “health provider networks,” or “patient networks” in conjunction with names of all countries categorized as LMICs according to the World Bank income groups classification for 2024–2025 (The World Bank 2024). A modified, updated search was run on PubMed, Embase (Ovid), SCOPUS, and PubMed Central in February 2025. We employed a flexible and iterative search strategy involving initial and pilot database searches, which helped expand the search terms and LMIC terminology, enabling the inclusion of all LMICs in the final search. Logical operator-based combinations of key terms were used. The final database search is presented in Supplementary File S2.

### Study selection and quality assessment

We screened the titles and abstracts of the retrieved articles against the eligibility criteria using the web application Rayyan (Ouzzani et al. 2016). After removing duplicates, the full text of the selected titles and abstracts was assessed against the eligibility criteria by H.O., H.B., and P.A. A second reviewer (D.D.G.) screened and reviewed a random sample of 20% of the titles and abstracts and the literature selected for full-text review. In case of disagreement, the team discussed the issues until a consensus was reached. A manual search was performed by D.D.G. to identify additional studies from the reference lists of relevant publications. Targeted searching was conducted in Google and Google Scholar. This was done by entering the full title of the text into the search engine and retrieving the full text, where available.

Methodological quality of the included studies was assessed using the ROBINS-I tools (Sterne et al. 2016) for assessing risk of bias in nonrandomised studies of interventions, while descriptive case studies consisting of qualitative and/or quantitative approaches were assessed using the Mixed Methods Appraisal Tool (MMAT) version 2018 (Hong et al. 2018) by N.S. and validated by D.D.G. The full assessments are presented in Supplementary Files S3 and S4.

### Charting the data

**Figure 1:**
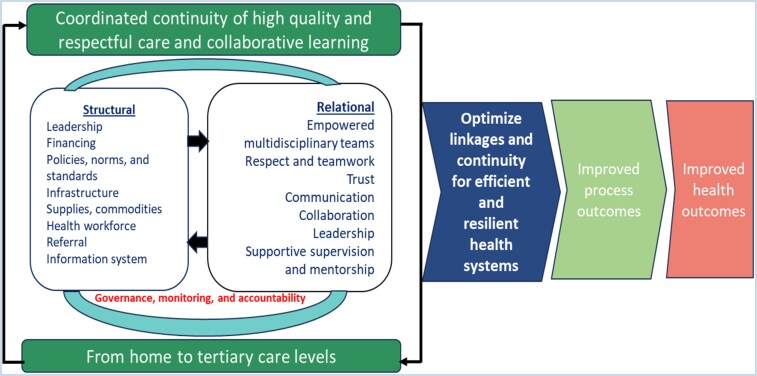
A logical model of the causal pathway of primary healthcare provider networks on improving healthcare outcomes (adapted from Kalaris et al. 2022).

We developed a data extraction tool in Excel to extract the data. The extraction was conducted by H.B., H.O., and P.A. and validated by D.D.G. We adapted the conceptual framework on the structural and relational aspects of networks of care (NOC) for maternal and newborn health by Kalaris et al. (2022) as the causal pathway logic model for our study (Fig. 1) to organize and explore the literature identified from the search. The framework suggests that a functional provider network, built on the existing structures within the health system and incorporating an improved relational element to enhance relationships, connections, and collaboration among cadres, facilities, and levels of care, will promote collaborative and coordinated continuity of high-quality, respectful care. This approach will optimize linkages for efficient and resilient health systems and ultimately lead to improved processes and health outcomes.

We extracted information on the author(s); year of publication; study title; country of study; study design; scope of the population (rural, urban or mixed); whether the network composition consists of only public facilities, private facilities or combination of both; name of the network; health issues of focus by the network; description of the network and the key findings on the process and clinical outcomes of the health services of focus. We resolved all disagreements through discussions until we reached a consensus. We discussed the results and continuously iteratively updated the data charting form.

### Collating, summarizing, and reporting the results

We applied a narrative synthesis to describe the included studies and their results. Through inductive and deductive processes, we extracted, coded, and categorized evidence from the included articles to identify the impact of PHCPNs on the process and clinical outcomes of the health services reported in LMICs. The data were coded against the causal pathway logic model of PHCPNs (Fig. 1). We defined the process outcomes based on the process elements—access, coverage, quality and safety—of the health systems building blocks framework by the World Health Organization (WHO) (World Health Organization 2010, Mounier-Jack et al. 2014) while the clinical outcome was used to represent the improved health component of the framework and was categorized into improved maternal mortality rate (MMR), improved under-5 (0–59 months) mortality, improved neonatal mortality rate (first 1 month of life), improved perinatal mortality, and improved stillbirths based on the emerging themes from the synthesis of the reviewed articles. The impacts of the networks were based on the reported outcomes by the individual papers. As it is the focus of this article, we only reported on the process and clinical outcomes of the health services provided by the networks. The charted data were mapped, similar data were grouped, and the frequency within each data group was reported. Upon completing each data extraction and analysis step, the team reviewed and discussed the findings. The findings are presented as evidence maps and in tables.

## Results

In this section, we will first present the results on the extent, range, and nature of research activity on PHCPNs in LMICs, then discuss the various types of PHCPNs identified in the literature, followed by discussions of process and clinical outcomes of PHCPNs reported by the included papers.

**Figure 2:**
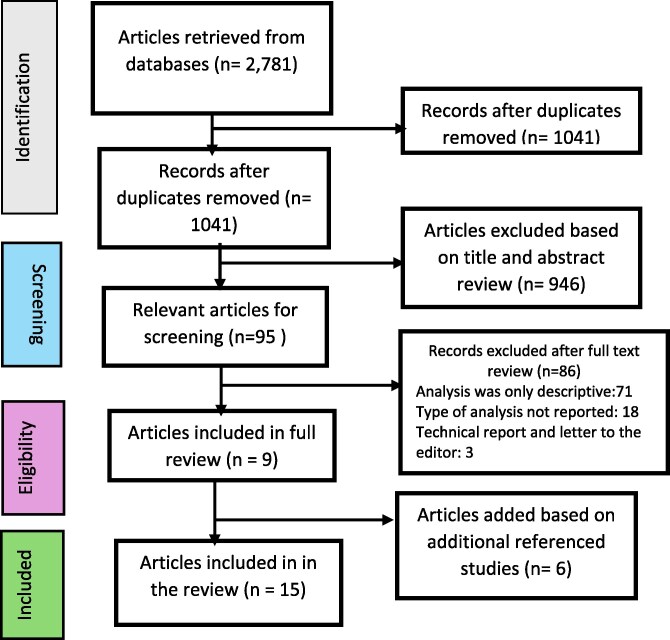
Flow diagram of study selection process.

Figure 2 illustrates the flow diagram of the selection process, detailing the total number of studies identified, those excluded, the reasons for their exclusion, and the studies ultimately included. Initially, 1041 potentially relevant studies were identified through electronic database searches and article reference reviews, following the removal of duplicates. After screening titles and abstracts, 946 studies were excluded because they merely mentioned networks without applying them as PHCPNs to achieve a health outcome. The remaining 95 full-text articles were retrieved and assessed for eligibility. It was observed that most of the identified studies were descriptive and did not systematically link process and clinical outcomes to the PHCPNs. Consequently, 86 out of the 95 articles were excluded for only providing a descriptive view of the networks, technical reports, and letters to the editor, without reporting the impact of the PHCPNs on any process and clinical outcomes. The remaining nine studies were deemed eligible for the study. Additionally, the updated searches retrieved six more pieces of literature that met the eligibility criteria, resulting in a total of 15 articles suitable for the review.

### Extent, range, and nature of published research on PHCPNs in LMICs

Table 2 presents the full list (*n* = 15) of the included studies in this review. The different papers examined different networks, with each paper primarily examining one type of network. Together, the articles reported on PHCPNs from eight (8) different countries. Most studies were reported from Indonesia (*n* = 4) (Ahmed et al. 2019, Hyre et al. 2019, Pedrana et al. 2019, Tholandi et al. 2019) and Madagascar (*n* = 4) (Garchitorena et al. 2017, 2018; Ezran et al. 2019, Cordier et al. 2020). The articles were largely recent, with publication dates ranging from 2017 to 2025. The highest number of publications was seen in 2019 (*n* = 5) (Ahmed et al. 2019, Ezran et al. 2019, Hyre et al. 2019, Pedrana et al. 2019, Tholandi et al. 2019) and 2020 (*n* = 5) (Bhatta et al. 2020, Cordier et al. 2020, Fasawe et al. 2020, Martinez Vergara et al. 2020, Sequeira D’Mello et al. 2020). This indicates the recent adoption of PHCPNs in LMICs. Most studies (*n* = 9) were evaluation studies, including quasi-experimental design using difference-in-difference (Garchitorena et al. 2018, Ezran et al. 2019, Tholandi et al. 2019), random effects poisson regression (Ahmed et al. 2019, Pedrana et al. 2019), pre- and post-evaluations (Sloan et al. 2018, Kamanga et al. 2022), interrupted time-series and generalized linear regression models (Garchitorena et al. 2017), and mixed-methods evaluation approaches (Tiruneh et al. 2025), while six were case studies (Hyre et al. 2019, Bhatta et al. 2020, Cordier et al. 2020, Fasawe et al. 2020, Martinez Vergara et al. 2020, Sequeira D’Mello et al. 2020). Most of the reviewed articles reported networks that focused solely on the rural population (*n* = 7) (Garchitorena et al. 2017, 2018; Ezran et al. 2019, Bhatta et al. 2020, Cordier et al. 2020, Kamanga et al. 2022, Tiruneh et al. 2025), while two focused on the urban population (Martinez Vergara et al. 2020, Sequeira D’Mello et al. 2020) and six on rural and urban mix (Sloan et al. 2018, Ahmed et al. 2019, Hyre et al. 2019, Pedrana et al. 2019, Tholandi et al. 2019, Fasawe et al. 2020). In terms of the health facility types that form the PHCPNs, eight of the reviewed articles were on PHCPNs that are public providers (Garchitorena et al. 2017, 2018; Sloan et al. 2018, Ezran et al. 2019, Bhatta et al. 2020, Cordier et al. 2020, Sequeira D’Mello et al. 2020, Kamanga et al. 2022) while seven reported on public–private providers (Ahmed et al. 2019, Hyre et al. 2019, Pedrana et al. 2019, Tholandi et al. 2019, Fasawe et al. 2020, Martinez Vergara et al. 2020, Tiruneh et al. 2025).

**Table 2:** Overview of included papers.

Authors (Year)	Study title	Country of study	Study design	Rural or urban (Scope of population)	Private, public
Tholandi et al. (2019)	The effect of expanding maternal and neonatal survival interventions on improving the coverage of labor monitoring and complication prevention practices in hospitals in Indonesia: a difference-in-difference analysis	Indonesia	Quasi-experimental design (difference-in-difference analysis)	Rural and urban	Public and private
Hyre et al. (2019)	Expanding maternal and neonatal survival in Indonesia: A program overview	Indonesia	A descriptive case study	Rural and urban	Public and private
Pedrana et al. (2019)	Assessing the effect of the Expanding Maternal and Neonatal Survival program on improving stabilization and referral for maternal and newborn complications in Indonesia	Indonesia	Evaluation (random-effects Poisson regression models)	Rural and urban	Public and private
Ahmed et al. (2019)	Changes in obstetric case fatality and early newborn mortality rates in hospitals after the implementation of the Expanding Maternal and Neonatal Survival program in Indonesia: Results from a health information systems	Indonesia	Evaluation (random-effects Poisson regression models)	Rural and urban	Public and private
Kamanga et al (2022)	Reducing maternal and neonatal mortality through integrated and sustainability-focused programming in Zambia	Zambia	Pre–post evaluation	Rural	Public
Fasawe et al (2020)	Applying a client-centered approach to maternal and neonatal networks of care: case studies from urban and rural Nigeria	Nigeria	A descriptive case study	Rural and urban	Public and public and private
Sloan et al. (2018)	Advancing survival in Nigeria: A pre-post evaluation of an integrated maternal and neonatal health program	Nigeria	Pre–post evaluation	Rural	Public
Bhatta et al. (2020)	The logarithmic spiral of networks of care for expectant families in rural Nepal: a descriptive case study	Nepal	Descriptive case study	Rural	Public
Cordier et al (2020)	Networks of care in rural Madagascar for achieving universal health coverage in Ifanadiana district	Madagascar	Descriptive case study	Rural	Public
Garchitorena et al. (2018)	Early changes in intervention coverage and mortality rates following the implementation of an integrated health system intervention in Madagascar	Madagascar	Evaluation (difference-in-difference analysis)	Rural	Public
Ezran et al (2019)	Assessing trends in the content of maternal and childcare following a health system strengthening initiative in rural Madagascar: a longitudinal cohort study	Madagascar	Evaluation (difference-in-difference analysis)	Rural	Public
Garchitorena et al (2017)	In Madagascar, use of health care services increased when fees were removed: lessons for universal health coverage	Madagascar	Interrupted time-series and generalized linear regression models	Rural	Public
D’Mello, et al. (2020)	Averting maternal death and disability in an urban network of care in Dar es Salaam, Tanzania: a descriptive case study	Tanzania	A descriptive case study	Urban	Public and privates
Martinez Vergara et al. (2020)	Building trust to save lives in a Metro Manila public-private network of care: a descriptive case study of Quirino Recognized Partners in Quezon City, Philippines	Philippines	A descriptive case study	Urban	Public and private
Tiruneh et al. (2025)	Networks of care for optimizing Primary Health Care Service Delivery in Ethiopia: Enhancing relational linkages and care coordination	Ethiopia	Mixed-method evaluation using RE-AIM	Rural	Public and private

RE-AIM, Reach, Effectiveness, Adoption, Implementation and Maintenance.

### Types of PHCPNs from the reviewed articles

Eight PHCPNs across different contexts and countries were identified in the papers reviewed (Table 2). They were the NOC of Indonesia (Ahmed et al. 2019, Hyre et al. 2019, Pedrana et al. 2019, Tholandi et al. 2019), Network of Safety in Nepal (Bhatta et al. 2020), Networks of Care of Madagascar (Garchitorena et al. 2017, 2018; Ezran et al. 2019, Cordier et al. 2020), Network of Care of Tanzania (Sequeira D’Mello et al. 2020), Network of Care of the Philippines (Martinez Vergara et al. 2020), Networks of Care of Nigeria (Sloan et al. 2018, Fasawe et al. 2020), Networks of Care of Zambia (Kamanga et al. 2022), and Networks of Care of Ethiopia (Tiruneh et al. 2025). The review shows that differences in the PHCPNs tend to be driven by the context, goal, and activities of the network (Table 3). For instance, although NOC as a PHCPN type was reported in seven out of the eight countries, much of the variation was due to the country of implementation, the objective, and specific activities involving different stakeholders based on the country context. For example, in Tanzania, the NOC involves urban public–private facilities including 22 government hospitals and catchment facilities operating across Dar es Salaam including 3 regional referral hospitals, 5 primary hospitals, 6 Comprehensive Emergency Obstetric and Newborn Care (CEmONC) health centres, and 8 dispensaries where lower-level facility staff were trained to recognize obstetric emergencies early, provide emergency resuscitation, and make referrals to a linked higher comprehensive emergency obstetric care (CEmOC) health facility through an enhanced referral system (Sequeira D’Mello et al. 2020) while the NOC in rural Ethiopia connected rural public–private PHC facilities and communities through administrative and clinical support mechanisms and implemented quality improvement activities to enhance relational linkages among the health workers and facilities, care coordination, strengthen referral linkages and improve the delivery of reproductive, maternal, newborn and child health (RMNCH) services and care outcomes at the woreda level (Tiruneh et al. 2025). Additionally, while Network of Safety in Nepal meets the definition of NOC (Bhatta et al. 2020), the network is termed ‘network of safety’ because of its goal, which was centered on the specific needs of the expectant women aimed to address the huge disparities in maternal survival between rural and urban Nepal.

**Table 3:** Networks and their descriptions.

Name of network (Country of implementation)	Health issues of focus	Problem	Description of the network from the reviewed papers	Location (rural or urban)	Focus in relation to PHC and ownership (1 All pubic, 2 all private, and 3 public private mix)
Network of care (Indonesia)	Maternal and newborn health	High maternal mortality (ratio of 305/100 000), influenced by poor obstetric care	The network was operationalized between 2011 and 2017 by the government of Indonesia in partnership with the United States Agency for International Development (USAID) to support the goal of reducing maternal and newborn mortality through the Expanding Maternal and Neonatal Survival (EMAS) intervention by strengthening the quality of care provided in community health centers (*puskesmas*) and hospitals. The key activities of the network were facility-based reviews of maternal and newborn deaths, peer-to-peer mentoring, health care skill improvement through in-service capacity strengthening, organization and communication of referrals, community engagements, and usage of data for decision making	Rural and urban	Public and private networks with care within community health centers and referral to higher levels
Network of safety (Nepal)	Maternal and newborn health	To address the reproductive needs of women living in remote areas of Nepal	The Network of Safety model was created in 2010 by One Heart Worldwide (OHW), a nongovernmental organization, in collaboration with the government health system in Nepal to address the reproductive health needs of women living in remote areas of Nepal, in collaboration with local-level health and government workers by emphasizing clinical skill development and mentorship in management and leadership. The network encompasses the family, the community, the village-level female community health volunteers (FCHVs), skilled providers, properly equipped health facilities, and the government system that supports it. In the network, each birthing center is connected to a group of FCHVs, a health facility, and secondary and tertiary facilities in a designated catchment area with clear roles and responsibilities	Rural	Public network with birthing centers and higher referral levels
Networks of care (Madagascar)	Maternal, newborn, and child health (MNCH)	Worse measures of mortality and care-seeking	This is a district-focused network created in 2014 by the Ministry of Public Health (MoPH) in partnership with PIVOT, a nongovernmental organization (NGO), to support high-quality patient-centerd care by integrating clinical care, improving patient referral, system readiness, and scientific innovation at all levels of care. The network comprises a referral supported by *Centre Hospitalier de Reference de District*-CHRD (public sector referral hospital), ambulance and stretcher deployment, referral documentation, community health sites, removal of patient fees for essential medication, an interactive dashboard to track the health system, maternal and perinatal death reviews, and a shift to community-based care from facility-based care. The network begins at the household level with a focus on community health, extends through primary and secondary care in the district, and on to tertiary care when needed, facilitating comprehensive quality care and linkages between levels of care for those with complex conditions	Rural	Public network with care within community health centers and referral to higher levels
Network of care (Tanzania)	Maternal and neonatal	Substandard quality of service leading to an excess of preventable maternal and neonatal morbidity and mortality	The network is a multifacility maternal and neonatal network developed originally, started in 2010 later upgraded in 2011, 2013 and 2020 by a nongovernmental organization, Comprehensive Community Based Rehabilitation in Tanzania (CCBRT) among 22 government hospitals and catchment facilities operating across Dar es Salaam including three regional referral hospitals, five primary hospitals (for maternity services), six Comprehensive Emergency Obstetric and Newborn Care (CEmONC) health centers, and eight dispensaries. In the network, the lower-level facility staff were trained to recognize obstetric emergencies early, provide emergency resuscitation, and make referrals to a linked higher CEmOC health facility through an enhanced NOC referral system	Urban	Public and private networks with dispensaries, health centers, and higher referral levels
Network of care (Philippines)	Maternal and newborn	Overcrowding and quality challenges in maternity wards resulting in high rates of maternal and neonatal death and near-miss	The network created in 2013 is a Quirino Recognized Partners (QRP) public–private partnership, uniting the public hospitals, some public health centers, and a host of private birth centers run by entrepreneurial midwives. The network adopts a hub-and-spoke model where the Quirino Memorial Medical Centre serves as the hub and 31 other facilities, mostly private midwife-owned clinics in six districts of Quezon City, Metro Manila, serve as spokes. The network focused on building trust through agreements with private and public birth centers within a 10-km radius, rolling out antenatal use of clinical and socioeconomic risk scoring tool, reaching out to potential clients and establishing clear communication channels and protocols for care and distribution of maternity cases by risk profile among the facilities and location. In the network, maternity cases are rationally distributed by risk profile and location among the facilities	Urban	Public and private network with health centers, birthing centers, and higher referral levels
Networks of care (Nigeria)	Maternal and newborn	Up to 90% home delivery, a high maternal mortality ratio and neonatal mortality rates, coupled with poor access and transportation to reach health care	The network created in 2014 is a partnership between the Government of Norway, the Federal Government of Nigeria, and the nongovernmental organization the Clinton Health Access Initiative (CHAI), focused on extending the reach of routine and emergency maternal and neonatal health services to reduce maternal and neonatal morbidity and mortality. These are two NOCs. One is a public facility (including registered TBAs) network focused on rural and semirural states of the state governments of Kaduna, Kano, and Katsina, while the other is a rural and periurban public–private network in Lagos state (linkages among registered traditional birth attendant clinics, private and public sector facilities, primary care board, and traditional medicine board). Though the two NOCs differ in setting, both provide pragmatic approaches to supporting women birthing in the community by integrating knowledge improvement and competency-based training for healthcare workers, mentor and equip traditional birth attendants-TBAs, continuous on-the-job mentoring, protocols and clear guides on management of clinical emergencies, expanded emergency transport scheme using volunteer taxi drivers, strengthened referral linkages and communication, use of motorbike ambulances (MBAs) at community level, develop state ambulance strategy, support large-scale roll out of low-cost, life-saving devices and commodities (Non-Pneumatic Anti Shock Garments—NASGs, manual neonatal resuscitator -MNRs etc), developed sustainable distribution system to ensure continuous commodity availability, reintroduce and strengthened community-based health management information system (CBHMIS), review reporting and link referral pathways from community up to tertiary hospital	Rural and urban	Public and also public-private networks with TBAs and higher referral levels
Networks of care (Zambia)	Sexual, reproductive, maternal and newborn health (SRMNH)	High lifetime risk of 1 in 100 of dying in pregnancy or childbirth and 1 in 37 infants die in their first month of life	The network was implemented between 2018 and 2021 across 141 government-owned health facilities covering all 12 districts of Northern Province as a collaboration between the Ministry of Health (MOH) of Zambia and the Clinton Health Access Initiative (CHAI) to reduce maternal, neonatal, and perinatal mortality by 40%, 40%, and 20%, respectively, over 4 years in the Northern Province of Zambia. The network creates and reinforces parallel and horizontal linkages across all levels of the health system, including implementing community-based activities through local stakeholders and referrals, and supporting facility-based activities such as training and mentoring, ensuring the availability of essential commodities and equipment, functional procurement and supply chain system, functioning and sustainable emergency transport, communication and referral systems, and robust management and information systems	Rural	Public network with care from community health centers and across all levels of care
Networks of Care (Ethiopia)	Reproductive, maternal, newborn, and child health (RMNCH)	Suboptimal quality care, fragmented and uncoordinated health care, and poorly functioning referral systems within PHC facilities	A collaboration between JSI and Amref Health Africa in 2022 to strengthen the functionality and bidirectional linkages across PHC delivery platforms using woreda (district)—wide NoCs. This involved designing and implementing strategies that connect public and private PHC facilities and communities through administrative and clinical support mechanisms and implementing quality improvement activities to enhance relational linkages among the health workers and facilities, care coordination, strengthen referral linkages and improve the delivery of reproductive maternal, newborn and child health (RMNCH) services and care outcomes at the woreda level. The network is characterized by clear agreements, supportive linkages, feedback loops, standards-based clinical skill-building, mentoring with routine case reviews, and joint learning forums	Rural	Public–Private network at the woreda (district) level

Though the papers we reviewed were not explicit about the configuration (i.e, hub and spoke or any other configuration) of the networks (only one indicated (Martinez Vergara et al. 2020)), they were clear about the reason for the establishment of the network (problem), the location (rural or urban), focus in relation to PHC, and the type of ownership (all public, all private, and public-private mix), and health services. All eight network types were implemented between 2010 and 2022 and were focused only on maternal, newborn, and child health. The problem they sought to address was high maternal and neonatal mortality and morbidity. Two PHCPNs were exclusively implemented in the urban localities (Martinez Vergara et al. 2020, Sequeira D’Mello et al. 2020). In terms of PHC focus and facility ownership, five of the PHCPNs were implemented as public-private networks (Hyre et al. 2019, Fasawe et al. 2020, Martinez Vergara et al. 2020, Sequeira D’Mello et al. 2020, Tiruneh et al. 2025), while four were public facility-only networks (Sloan et al. 2018, Ezran et al. 2019, Bhatta et al. 2020, Kamanga et al. 2022). None of the PHCPNs was implemented as a private facility-only network. However, all the networks were focused on bringing services closer to the community and facilitating referrals from the community level to the higher level of care. The descriptions of each network are provided in Table 3.

### Impacts of PHCPNs on process and clinical outcomes of primary health care services

Although the articles we reviewed focused on specific networks, they also provided information about the broader context in which the networks existed. They also had information on process outcomes that can be categorized under the six WHO health systems building blocks (World Health Organization 2010, Mounier-Jack et al. 2014) and on health outcomes measured by impacts on morbidity and mortality. The eight PHCPNs, irrespective of whether they were implemented in rural and urban or mixed contexts, were comprised of public, private, or mixed public–private ownership, documented contributions to improvements in the process outcomes of health services by enhancing access to care (Tholandi et al. 2019, Fasawe et al. 2020, Martinez Vergara et al. 2020, Tiruneh et al. 2025), coverage of health services (Garchitorena et al. 2018, Cordier et al. 2020, Kamanga et al. 2022), quality of care and services (Garchitorena et al. 2018, Tholandi et al. 2019, Cordier et al. 2020, Martinez Vergara et al. 2020, Kamanga et al. 2022, Tiruneh et al. 2025), and safety of care (Tholandi et al. 2019), and in clinical outcomes by helping to reduce maternal (Sloan et al. 2018, Hyre et al. 2019, Pedrana et al. 2019, Tholandi et al. 2019, Bhatta et al. 2020, Martinez Vergara et al. 2020, Sequeira D’Mello et al. 2020, Kamanga et al. 2022, Tiruneh et al. 2025), neonatal (Garchitorena et al. 2018, Sloan et al. 2018, Ahmed et al. 2019, Pedrana et al. 2019, Bhatta et al. 2020, Martinez Vergara et al. 2020, Kamanga et al. 2022), and perinatal (Sloan et al. 2018, Sequeira D’Mello et al. 2020, Kamanga et al. 2022, Tiruneh et al. 2025) mortalities and stillbirths (Sloan et al. 2018) (Table 4).

**Table 4:** Findings on process outcomes of PHCPN implementation.

Improved access to care	Improved service coverage	Quality of care and services	Improved safety
Improved referral effectiveness (Tholandi et al. 2019)	Improved coverage for sexual, reproductive, maternal, and newborn health (SRMNH) services (Kamanga et al. 2022)	Improvement in the treatment of newborns with suspected severe infection (Tholandi et al. 2019)	Better performance of labor monitoring (14 points higher) (Tholandi et al. 2019)
Facilitated rapid referrals (Fasawe et al. 2020)	Significant improvements in the coverage at facilities and improvement in Integrated Management of Childhood Illness (IMCI) at community and CSB levels, malnutrition and tuberculosis (Cordier et al. 2020)	Improved service readiness for sexual, reproductive, maternal, and newborn health (SRMNH) services (Kamanga et al. 2022)	Better performance of newborn resuscitation readiness (38 points higher) (Tholandi et al. 2019)
Increased access to vaccinations, uptake of FP services, and HIV screening (Fasawe et al. 2020)	Increased coverage of health services (Bhatta et al. 2020)	Reduction in prolonged patient waiting time (Martinez Vergara et al. 2020)	Improved reduction in perinatal asphyxia (though not statistically significant) (Martinez Vergara et al. 2020)
Improved referral turnaround time (Martinez Vergara et al. 2020)	Increase in MNCH coverage by 30.1% in health facilities (Garchitorena et al. 2018)	Improved reduction in perinatal asphyxia (though not statistically significant) (Martinez Vergara et al. 2020)	Better performance of infection prevention practices (33 points higher) (Tholandi et al. 2019)
Improved care coordination (Tiruneh et al. 2025)		Significant improvements in the quality of care at facilities (Cordier et al. 2020)	Increase in provider confidence in performing clinical procedures and responding to emergencies (Tholandi et al. 2019)
		The rate of ANC 8 + visits was 29.8% per month higher than expected without the NOCs strategy (Coef: 2.39; *P*-value < 0.01) (Tiruneh et al. 2025)	
		63% increase in deliveries in health facilities (Garchitorena et al. 2018)	

FP, family planning; MNCH, maternal, newborn and child health.

### Impacts of PHCPNs on process outcomes of health services

#### Improved access to care

Four of the identified PHCPNs in the reviewed articles (Table 4) reported the impact on access to care (Tholandi et al. 2019, Fasawe et al. 2020, Martinez Vergara et al. 2020, Tiruneh et al. 2025). The studies demonstrated that PHCPNs improved access to care by enhancing referral effectiveness (Tholandi et al. 2019), facilitating rapid referrals (Fasawe et al. 2020), increasing access to vaccination, uptake of family planning services, and HIV screening (Fasawe et al. 2020). Further synthesis showed that PHCPNs improved the turnaround time through an enhanced referral and communication system for maternal and neonatal services with an average transfer time of 45 minutes, which was considered a short turnaround time in Metro Manila (Martinez Vergara et al. 2020), and enhanced care coordination (Tiruneh et al. 2025). Also, the public PHCPN in rural Madagascar reported a 51% increase in care seeking among children with diarrhea, persistent cough, and fever 2 years following its implementation, while non-network districts recorded a decrease of 7%. Moreover, the network recorded a 52% rise in service utilization by children under 5, and over 25% growth in maternal consultations following the implementation of exemptions for targeted medicines and point-of-service fees (Garchitorena et al. 2018). In northern Nigeria, the PHCPN recorded improved access to maternal and newborn services by facilitating rapid referrals while contributing to 7% increase in facility use (Fasawe et al. 2020).

#### Improved service coverage

Four of the PHCPNs in the reviewed papers reported improved coverage of health services (Garchitorena et al. 2018, Cordier et al. 2020, Kamanga et al. 2022). The review highlighted enhanced coverage of sexual, reproductive, maternal, and newborn health (SRMNH) through PHCPN in Zambia through reinforcement of parallel and horizontal linkages across all levels of the health system, including implementing community-based activities through local stakeholders, supporting facility-based activities such as training and mentoring, ensuring the availability of essential commodities and equipment, functioning and sustainable emergency transport, communication and referral systems, and robust management and information systems (Kamanga et al. 2022). Additionally, significant improvements in the coverage and improvement in Integrated Management of Childhood Illness (IMCI) were reported at the community and CSB (*Centre de Santéde Base*) levels by PHCPN in rural Madagascar through the integration of clinical care, enhanced patient referral, and system readiness (Cordier et al. 2020). The evaluation of the PHCPN demonstrated a significant increase in coverage from 49.3% to 64.1% compared to 34.2% to 44.9% observed in non-networked districts for maternal, newborn, and child healthcare services. Furthermore, the network recorded a 20% increase in coverage of curative care for children under 5 (Ezran et al. 2019). Similarly, in rural Nepal, heightened coverage of health services for expectant families was reported following the implementation of PHCPN, where clinical skills development and mentorship in management and leadership were better enhanced (Bhatta et al. 2020), while service coverage for maternal and neonatal, and child health (MNCH) in Indonesia increased by 30.1% due to PHCPN (Garchitorena et al. 2018).

#### Improved quality of care and services

In terms of quality of care and services, five of the networks reported in the reviewed articles demonstrated impacts (Garchitorena et al. 2018, Tholandi et al. 2019, Cordier et al. 2020, Martinez Vergara et al. 2020, Kamanga et al. 2022, Tiruneh et al. 2025). The review showed that PHCPNs contribute to the improvement in the treatment of newborns with suspected severe infection in Indonesia when peer mentorship, healthcare skill, and referral were enhanced (Tholandi et al. 2019), improved service readiness for sexual, reproductive, maternal, and newborn health (SRMNH) services in Zambia as a result of functional procurement and supply chain system, functioning and sustainable emergency transport, communication and referral systems (Kamanga et al. 2022), and the reduction in prolonged patient waiting time in a Metro Manila Public-Private NoC in the Philippines through clear communication channels and protocols for care and distribution of maternity cases by risk profile among the facilities and locations (Martinez Vergara et al. 2020). In rural Madagascar, a 63% increase in health facility deliveries was recorded by the public PHCPN (Cordier et al. 2020). Additionally, the public–private PHCPN in rural Ethiopia recorded a 29.8% increase in ANC 8+ visits in a month, higher than expected without the network as a result of supportive linkages, feedback loops, standards-based clinical skill-building, mentoring with routine case reviews, and joint learning forums (Coef: 2.39; *P*-value < 0.01) (Tiruneh et al. 2025). Also, in Indonesia, 24% to 61% improvement was observed in stabilization practices of prereferral for pre-eclampsia, while treatment of newborns with suspected severe infection improved from 30% to 54% (Tholandi et al. 2019). Similarly, the rural public networks of safety in Nepal were found to contribute to improved quality of care, which resulted in improved utilization (more than 30%) of ANC services (Bhatta et al. 2020), while the public–private PHCPN in Tanzania improved the quality of care and services by adopting a strategy where the senior health workers constantly follow up on colleagues to offer assistance in providing care.

#### Improved safety of care

For safety of care, an improvement was reported by the reviewed papers in three of the PHCPNs (Tholandi et al. 2019, Fasawe et al. 2020, Martinez Vergara et al. 2020). Synthesis of the papers showed improved safety of pregnant women and newborns by the public PHCPN in rural Nepal due to increased provider confidence in performing clinical procedures and responding to emergencies (Bhatta et al. 2020). Additionally, the review highlights improved safety for pregnant women and newborns by enhancing the performance of labor monitoring (14 points higher), newborn resuscitation readiness by 38 points higher, and infection prevention practices following the implementation of public–private PHCPN in rural and urban Indonesia (Tholandi et al. 2019). Further synthesis of the reviewed articles showed that PHCPN helped in strengthening the health system by improving operational standards and promoting institutional deliveries, emergency transport systems, and clinical skills through training and mentoring, thereby ensuring timely access to appropriate care and safe services, spotting high-risk pregnancies early and referrals in Nigeria (Fasawe et al. 2020).

### Impacts of PHCPNs on clinical outcomes of health services

#### Maternal mortality

In terms of maternal mortality, seven of the PHCPNs demonstrated a significant reduction in maternal mortality (Garchitorena et al. 2018, Sloan et al. 2018, Ahmed et al. 2019, Pedrana et al. 2019, Bhatta et al. 2020, Martinez Vergara et al. 2020, Kamanga et al. 2022). For example, a 37% (OR 0.629, 95% CI 0.490–0.806) reduction in maternal mortality was reported following 18 months into the implementation of the PHCPN in rural northern Nigeria (Sloan et al. 2018). Additionally, in rural and urban Indonesia, evaluation of the public-private PHCPNs showed a 50% reduction in the case fatality rate from any maternal complications (Hyre et al. 2019, Pedrana et al. 2019, Tholandi et al. 2019), while in northern Zambia, a statistically significant decline of 41% in MMRs was recorded, over 4 years (between 2017 and 2021), following the PHCPN implementation (Kamanga et al. 2022). Also, the public–private rural–urban PHCPN in Indonesia recorded a 50% (from 5.5 to 2.6 deaths per 1000 cases of admitted obstetric cases) decline in case fatality rate among pregnant women with complications admitted to the network facility within 21–45 months after the operationalization of the network (Ahmed et al. 2019, Hyre et al. 2019, Pedrana et al. 2019, Tholandi et al. 2019). Furthermore, the public PHCPN in rural Nepal, where each birthing centre was connected to a group of FCHVs, a health facility, and secondary and tertiary facilities in a designated catchment area with clear roles and responsibilities, found an 80% reduction in maternal deaths (Bhatta et al. 2020). The review also noted a 49% [(35/100 000) compared to (68.7/100 000)] reduction in maternal mortality in the Metro Manila public-private PHCPN in Quezon City, the Philippines (Martinez Vergara et al. 2020).

#### Under-5 mortality (0–59 months)

Only one of the reviewed articles reported on under-5 mortality (Garchitorena et al. 2018). While the study reported a 19.1% decrease in under-5 mortality, the result was not statistically significant when compared with 14.9% in non-networked districts (Garchitorena et al. 2018).

#### Neonatal mortality (first month of life)

In terms of neonatal mortality, six of the PHCPNs from the reviewed papers (Table 5) reported a significant reduction in neonatal mortality (Garchitorena et al. 2018, Sloan et al. 2018, Ahmed et al. 2019, Pedrana et al. 2019, Bhatta et al. 2020, Martinez Vergara et al. 2020, Kamanga et al. 2022). The review showed that in rural northern Nigeria, the PHCPN contributed to a 43% (OR 0.574, 95% CI 0.503–0.655) reduction in neonatal mortality following 18 months of its implementation (Sloan et al. 2018). Similarly, a statistically significant decline of 45% in neonatal mortality rates was reported between 2017 and 2021 following the implementation of the public PHCPN in rural northern Zambia (Kamanga et al. 2022). The PHCPN in Indonesia reported a reduction of 21% (IRR 0.79; 95% CI, 065–0.96) in very early neonatal mortality rate (deaths within 24 hours of birth) (Ahmed et al. 2019, Pedrana et al. 2019). Also, the public PHCPN in rural Nepal found an 80% reduction in newborn deaths (Bhatta et al. 2020).

**Table 5:** Findings on health outcomes of PHCPN implementation.

Authors (Year)	Context/type of network	Study design	Rural or urban (Scope of population)	Ownership (1 All pubic, 2 all private, 3 public private mix)	Results—clinical outcomes (95% CI)				
					Improved maternal health	Improved under-5 (0–59 months) mortality	Improved neonatal mortality (First one month of life)	Improved perinatal mortality	Improved stillbirths
Tholandi et al. (2019)	Network of care (Indonesia)	Quasi-experimental design (difference-in-difference analysis)	Rural and urban	Public and private	CFR from any maternal complication decreased by 50% in hospitals CFR from any maternal complication decreased by 50% in hospitals CFR from any maternal complication decreased by 50% in hospitals N/A	N/A	N/A	N/A	N/A
Hyre et al. (2019)	Network of care (Indonesia)	Descriptive case study	Rural and urban	Public and private		N/A	N/A	N/A	N/A
Pedrana et al. (2019)	Network of care (Indonesia)	Evaluation (random-effects Poisson regression models)	Rural and urban	Public and private		N/A	Very early NMR decreased by 21%	N/A	N/A
Ahmed et al. (2019)	Network of care (Indonesia)	Evaluation (random-effects Poisson regression models)	Rural and urban	Public and private		N/A	Very early NMR decreased by 21%	N/A	N/A
Kamanga et al. (2022)	Networks of care (Zambia)	Pre–post evaluation	Rural	Public	MMR decreased 41%	N/A	NMR decreased by 45%	Perinatal mortality rate decreased by 43%	N/A
Fasawe et al. (2020)	Networks of care (Nigeria)	Descriptive case study	Rural and urban	Public and Public and private	N/A	N/A	N/A		
Sloan et al. (2018)	Networks of care (Nigeria)	Pre–post evaluation	Rural and urban	Public	MMR decreased 37% (OR 0.629, 95% CI 0.490–0.806) vrs baseline 440/100 000 births		NMR decreased 43% (OR 0.574, 95% CI 0.503–0.655) vrs baseline 15.2/1000 births	Perinatal mortality rate decreased 27% (OR 0.733, 95% CI 0.676–0.795) vrs baseline 36.0/1000 births	Stillbirth rate decreased 15% (OR 0.850, 95% CI 0.768–0.941) vrs baseline 21.1/1000 births
Bhatta et al. (2020)	Network of safety (Nepal)	Descriptive case study	Rural	Public	80% reduction in maternal	N/A	80% reduction in newborn deaths	N/A	N/A
Cordier et al (2020)	Networks of care (Madagascar)	Descriptive case study	Rural	Public	N/A	N/A	N/A	N/A	N/A
Garchitorena et al. (2018)	Networks of care (Madagascar)	Evaluation (difference-in-difference analysis)	Rural	Public	N/A	19.1% decrease in under-5 mortality, although not statistically significant	36.4% decrease in neonatal mortality, although not statistically significant	N/A	N/A
Ezran et al. (2019)	Networks of care (Madagascar)	Evaluation (difference-in-difference analysis)	Rural	Public	N/A	N/A	N/A	N/A	N/A
Garchitorena et al. (2017)	Networks of care (Madagascar)	Interrupted time-series and generalized linear regression models	Rural	Public	N/A	N/A	N/A	N/A	N/A
Sequeira D’Mello et al. (2020)	Network of care (Tanzania)	A descriptive case study	Urban	Public and privates	Reduction in maternal death rate by 40%	N/A	N/A	Decline in facility’s perinatal death rates	N/A
Vergara et al. (2020)	Network of care (Philippines)	A descriptive case study	Urban	Public and private	49% decline in maternal mortality in the network compared to Quezon City [(35/100 000) compared to (68.7/100 000)	N/A	Neonatal outcomes improved (decreased from 44.3/1000 to 32/1000), though not statistically significant	N/A	N/A
Tiruneh et al. (2025)	Networks of care (Ethiopia)	Mixed-method evaluation using RE-AIM	Rural	Public and private	18.4% increase in obstetric complications managed (Coef: 1.71; *P*-value = 0.050)	N/A	N/A	Perinatal mortality decreased by 34%, from 31.3 to 20.1 per 1000 births [*t*-test: 2.12; *P*-value: 0.040)]	N/A

MMR, maternal mortality rate; CFR, case fatality rate; OR, odds ratio.

However, some reductions in neonatal mortality were reported in the reviewed papers for the public PHCPN in rural Madagascar (Garchitorena et al. 2018) and the Philippines (Martinez Vergara et al. 2020), but they were not statistically significant.

#### Perinatal mortality

The reviewed papers reported statistically significant reductions in perinatal mortality by 4 out of the 10 identified PHCPNs (Sloan et al. 2018, Sequeira D’Mello et al. 2020, Kamanga et al. 2022, Tiruneh et al. 2025). The review showed that the PHCPN in northern Nigeria contributed to a 27% (OR 0.733, 95% CI 0.676–0.795) reduction in perinatal mortality (Sloan et al. 2018) while in northern Zambia, the contribution to the reduction in perinatal mortality was 43% (Kamanga et al. 2022). Additionally, the review showed that the public–private PHCPN in rural Ethiopia resulted in a 34% decline in perinatal mortality from 31.3 to 20.1 per 1000 births [*t*-test: 2.12; *P*-value: 0.040] (Tiruneh et al. 2025). Also, the public PHCPN in urban Tanzania reported a significant decline in perinatal death rates (Sequeira D’Mello et al. 2020).

#### Stillbirths

Stillbirth was reported by only one of the reviewed papers. The results showed the PHCPN in northern Nigeria contributed to a 15% (OR 0.850, 95% CI 0.768–0.941) reduction in stillbirth rates (Sloan et al. 2018).

## Discussion

The review conducted a systematic search of the literature to identify and map relevant primary studies, thereby establishing a comprehensive evidence based on the current use of PHCPNs, their various forms, and their potential effects on enhancing health systems in LMICs. The review provides a foundation for such future primary empirical research. To the best of our knowledge, this is the first systematic review on the impact of PHCPNs on process and clinical outcomes of health services in LMICs. Given the limited number of empirical studies found, there is significant potential for further research on PHCPNs, particularly in other areas of health services. The paper therefore adds to the literature that documents PHCPNs and their role in PHC implementation.

This systematic review of types of PHCPNs in LMICs and their impact on health systems processes and clinical outcomes offers several useful insights to inform policy and program design and implementation.

First, despite the limited size of the low and middle income country literature; there is quite a bit of emerging evidence that (i) PHCPNs contribute to improvements in the process outcomes of health services by enhancing access to care, coverage of health services, quality of care and services, and safety of care, and (ii) they support improvements in clinical outcomes by helping to reduce maternal, neonatal, and perinatal mortalities and stillbirths. The reported impacts of PHCPNs were observed across different PHCPN types (public, private, or mixed public-private facility ownership) and country contexts, including rural and urban. There is therefore empirical support for continued work on designing, implementing, and evaluating the role of provider networks in PHC in LIMC.

The review shows that PHCPNs primarily focus on maternal, newborn, and child health programs. This limits the broader understanding of the actual and potential impact of PHCPNs in other aspects of the disease burden in LMIC, such as communicable and non-communicable diseases. This is a major gap given the clear evidence of the growing dual burden of communicable and noncommunicable disease in LMIC as well as the needs of other demographic groups alongside the continuing vulnerability of mothers, babies, children, and adolescents. Future research on PHCPN in LMIC needs to look at the impact beyond MNCAH.

However, the findings suggest that the adoption of PHCPNs is of greater significance in advancing health system goals and UHC. The results confirmed existing evidence, which shows that integrating health services is key to achieving UHC (Marston et al. 2016, WHO 2017). For instance, the review showed that in rural Madagascar, where PHCPN was implemented with focus on integrating clinical care, improving patient referral, system readiness, and scientific innovation at all levels of care from the household level through primary and secondary to tertiary care, there was a significant improvement in the coverage and Integrated Management of Childhood Illness (IMCI) at the community and CSB (*Centre de Santéde Base*) levels (Cordier et al. 2020). Additionally, in northern Nigeria, a resource-constrained setting with high rates of maternal, perinatal, and newborn mortality, it was observed that the short-term impact of adopting a coherent, integrated approach through PHCPN, in improving maternal, perinatal, and newborn mortality is comparable to those achieved globally in the previous 15–20 years (Sloan et al. 2018). Reductions in maternal, neonatal, and perinatal mortalities remain key priorities under Sustainable Development Goal (SDG) 3, which aims to ensure healthy lives and promote well-being for all. The findings thus confirm the role of PHCPN in reaching this goal (United Nations 2020).

Our review also showed that PHCPNs exist in diverse contexts, ranging from rural, urban, and rural-urban mix, and across public and public–private provider mix and geographical regions. However, only two of the networks focused solely on the urban population. However, the approach of working in primary care teams has been presented as an important strategy in improving primary care in urban areas in LMICs (Lilford et al. 2025). In LMICs, urban populations are grappling with rapid and uncontrolled urbanization coupled with substantial barriers to quality and accessible primary healthcare (Mundia and Murayama 2010, Ezeh et al. 2017, Lilford et al. 2025). The infrastructure of these cities, particularly public primary care facilities, is largely ill-equipped to meet the evolving health needs of the large and expanding urban population and change in disease burdens (Adams et al. 2018, Elsey et al. 2019, Lilford et al. 2025). While the private sector (both formal and informal) has expanded to fill in the gaps left by the public sector, there remain limited to no linkages among facilities and concerns about the quality of care (Miller and Goodman 2016). The review demonstrated the benefits of PHCPNs, including quality of care, underscoring the need for urgent prioritization of PHCPNs as part of the health systems in urban LMICs. The limited PHCPNs in the urban context could be an indication of possible challenges to their adoption. While PHC in the rural context is predominantly public facilities, making implementation of PHCPNs fairly straightforward, urban PHC is often complex, with a plurality of public and private providers (Lilford et al. 2025).

Additionally, the review shows that differences in PHCPNs tend to be driven by the context, goal, and activities of the network. For instance, NOC as a PHCPN type was reported in seven out of the eight countries and tends to be driven by the context, goal, and activities in the different countries. Given that PHC is a whole-of-society approach to maximize the level and distribution of health and well-being, the findings suggest that PHCPN presents as an important approach in strengthening PHC in improving health for diverse populations in varied contexts. For example, the multifacility maternal and neonatal public–private PHCPN in the urban setting of Tanzania improved the quality of care and services (Sequeira D’Mello et al. 2020), while PHCPN in rural Ethiopia that connected public–private PHC facilities and communities through administrative and clinical support mechanisms strengthened referral linkages and improved the delivery of reproductive, maternal, newborn, and child health (RMNCH) services and care outcomes at the woreda level, including a significant increase in ANC 8+ visits (Tiruneh et al. 2025). Additionally, the public–private PHCPN in rural and urban Indonesia improved stabilization practices of prereferral for pre-eclampsia and treatment of newborns with severe infection (Tholandi et al. 2019). These findings suggest that PHCPNs have the potential to reinforce the tenets of PHC and improve health system outcomes in LMICs. The review serves as a mapping and clarification exercise to support the adoption of PHCPNs for strengthening health systems in LMICs.

Our review further indicates that the causal pathway logic model for our study (Fig. 1) could be a useful tool in the study of PHCPNs in other health service areas beyond maternal and child health, particularly in providing an understanding of how PHCPNs built on existing structures within the health system and incorporating an improved relational element to enhance relationships, connections, and collaboration among cadres, facilities, and levels of care, could improve process outcomes including access to care, coverage of health services, quality of care and services and safety of care, and improvements in clinical outcomes. We applied the model across different PHCPNs and contexts. The model provided descriptions of the different processes and clinical outcomes and an understanding of the mechanisms that underpinned the achievement of the particular impacts, suggesting that it offers value in generating policy-relevant theoretical insights about networks and their influences, particularly in LMICs. Moreover, the application of the model was within several research designs, implying the flexibility of the model in evaluating varied PHCPN types.

### Limitations of the study

The review has some limitations. Our search strategy was limited to LMICs, and the selected literature was limited to English due to the authors’ capabilities, potentially excluding relevant literature in other languages. This approach could have missed relevant learnings. Despite these limitations, the available evidence retrieved was able to provide an answer to the review question. We could not compare our findings to evidence in high-income countries (HICs). Though the inclusion of primary care networks in HIC contexts could enable transfer of knowledge across LMIC and HIC contexts, the objective of the paper was not to understand implementation experiences across contexts (which is beyond the scope of this study) but rather to explore the literature to provide an in-depth LMIC context-specific understanding of the impact of PHCPNs in terms of process and clinical outcomes to contribute to policy and program design and implementation of PHCPNs in LMIC.

## Conclusions

This body of literature we reviewed suggests that PHCPNs make a difference in the process and clinical outcomes of health services in LMICs. This review serves as both a mapping and clarification exercise to promote the adoption of PHCPNs and as a foundation for further research, especially in areas of health services beyond maternal, newborn, and child health.

## Supplementary Material

czag003_Supplementary_Data

## Data Availability

No data was used for the research described in the article.

## Reflexivity statement

The group of authors included five females and four males. The group comprised researchers based in Ghana and the UK, with multiple levels of seniority from predoctoral researchers to postdoctoral research fellow, associate, and full professors. All authors have extensive experience in health systems and policy research. The authors’ areas of study varied and include health system strengthening, analysis of health policy and its implementation in LMIC health systems, health economics, urban health systems, implementation science, and reviews. These different experiences and perspectives were harnessed and brought to bear on the work reported in this paper.
