# Supplementary material for: Primary health care networks and impacts in low- and middle-income countries: a systematic review
Source: Health Policy Plan. 2026 Jan 16;41(3):471–91. doi: 10.1093/heapol/czag003 (PMC12972678; doi:10.1093/heapol/czag003)
Supplement: czag003_Supplementary_Data [file czag003_supplementary_data.zip › Supplementary file 2.docx]

**Final search term and variations**

| **Main term** | **Variations of the term searched** |
| --- | --- |
| PHCPNs | primary healthcare provider networks; primary care networks; networks of practice; networks of care; care networks; health facility networks; health provider networks; patient network |
| Low and middle-income countries | Africa, developing countries, less developed countries, underdeveloped countries, low-and-middle-income countries, underserved communities, deprived communities, poor communities, Afghanistan, Burkina Faso, Burundi, Central African Republic, Chad, Congo, Democratic Republic of Congo, Eritrea, Ethiopia, Gambia, Guinea-Bissau, Korea Democratic People’s Republic, Liberia, Madagascar, Malawi, Mali, Mozambique, Niger, Rwanda, Sierra Leone, Somalia, South Sudan, Sudan, Syria, Togo, Uganda, Yemen, Angola, Bangladesh, Benin, Bhutan, Bolivia, Cabo Verde, Cambodia, Cameroon, Comoros, Cote d-Ivoire, Djibouti, Egypt, Eswatini, Ghana, Guinea, Haiti, Honduras, India, Jordan, Kenya, Kiribati, Kyrgyz Republic, Lao PDR, Lebanon, Lesotho, Mauritania, Micronesia, Morocco, Myanmar, Nepal, Nigeria, Pakistan, Papua New Guinea, Philippines, Samoa, Sao Tome and Principe, Senegal, Solomon Islands, Sri Lanka, Tajikistan, Tanzania, Timor-Leste, Uzbekistan, Vanuatu, Vietnam, West Bank and Gaza, Zambia, Zimbabwe |
