# Supplementary material for: Primary health care networks and impacts in low- and middle-income countries: a systematic review
Source: Health Policy Plan. 2026 Jan 16;41(3):471–91. doi: 10.1093/heapol/czag003 (PMC12972678; doi:10.1093/heapol/czag003)
Supplement: czag003_Supplementary_Data [file czag003_supplementary_data.zip › Supplementary file 3.docx]

**ROBINS-1 Quality assessment**

| N | **Study** | **Bias due to confounding** | **Bias in selection of participants into the study** | **Bias in classification of interventions** | **Bias due to deviations from intended interventions** | **Bias due to missing data** | **Bias in measurement of outcomes** | **Bias in selection of the reported result** | **Final classification** |
| --- | --- | --- | --- | --- | --- | --- | --- | --- | --- |
| 1 | Sloan, 2018 | Serious: uncontrolled baseline confounding | Low: Entire populations in selected states were tracked via key informants , no indication of selection unrelated to eligibility. | Low: Intervention timing was well defined | Low: intervention was implemented throughout; no deviations or co-interventions are described | No information: No discussion of missing or incomplete data | Moderate: Vital events recorded via community key informants that can vary in accuracy | Low: Mortality outcomes were reported with odds ratios and regression analyses | Serious |
| 2 | Ahmed et al. (2019) | Moderate: No control group | Moderate: participants selected from hospitals where EMAS was implemented, excluding non-participating facilities | Low: Implementation and timing of EMAS were clearly defined across phases | Low: No reported deviations | Low: Data quality was assessed via RDQA tools in 2013, 2015, and 2016 | Low: Mortality outcomes based on routine registers | Moderate: focused on CFR and early neonatal mortality; other relevant outcomes (e.g., longer-term neonatal mortality, referral processes) were not reported | Moderate |
| 3 | Tiruneh, et al. (2025) | Moderate: it lacks an external comparison group | Moderate: Facility selection may be non-random | Low: NoCs approach clearly defined, structured implementation using RE-AIM. | Low: Framework adaptation is described | Moderate: routine data can lead to incomplete coverage or data quality issues | Moderate: Quantitative outcomes via ITS are robust, but qualitative interpretation can involve subjectivity | Moderate: Focused reporting on positive outcomes | Moderate |
| 4 | Ezran et al (2019) | Moderate: lack of random assignment can lead to unmeasured confounding | Low: Households were district-representative | Low: Intervention definition is clear and based on geographic catchment of HSS program | Low: No major deviations reported | Moderate: Self-reported care. Non-response or recall bias can affect data completeness | Moderate: Dif in Dif analysis is a strength, but self-reported measures can introduce misclassification or recall bias | Moderate: focused on positive improvements. Broader or null findings can be underreported. | Moderate |
| 5 | Pedrana, et al. (2019) | Moderate: lack of random allocation can lead to unmeasured confounding | Moderate: Facilities receiving EMAS support can differ systematically from comparison facilities in terms of infra-structure and quality of the services | Low: EMAS intervention Cleary defined | Low: No major deviations reported | Low: Based on routine monitoring data, data completeness and consistency are not fully reported | Low: Stabilisation rates and referral system metrics reduce the risk of bias | Moderate: focused on positive improvements. Broader or null findings can be underreported. | Moderate |
| 6 | Kamanga, et al (2022) | Moderate: No comparison group, trends or concurrent interventions can influence outcomes | Moderate: intervention areas could be selected due to high need or support readiness | Low: Intervention Cleary defined, based on six health system pillars and aligned with national systems | Low: No major deviations reported. Programme designed for sustainability and integrated into existing government systems | Moderate: Based on routine health systems and community surveillance data, data completeness and consistency are not fully reported | Moderate: Reliance on HMIS and early surveillance can miss events or be influenced by reporting biases | Moderate: focused on positive improvements. Broader or null findings can be underreported. | Moderate |
| 7 | Tholandi, et al (2019) | Moderate: lack of random allocation can lead to unmeasured confounding | Moderate: Hospitals in intervention arm can differ in terms of better resources and other characteristics | Low: Well-defined exposure and clear demarcation between EMAS and comparison hospitals | Low: No major deviations reported. Direct observation leading to good fidelity | Low: Direct observation minimises data gap | Low: Outcomes are directly observed | Moderate: focused on positive improvements. Broader or null findings can be underreported. | Moderate |
| 8 | Garchitorena, et al. (2018) | Moderate: lack of random allocation can lead to unmeasured confounding | Low: The district-representative cohort reduces selection bias | Low: The HSS zone is clearly defined geographically | Low: No major deviations reported | Moderate: Household surveys can include non-response or recall bias | Moderate: Coverage indices and mortality estimates derived from survey data, mortality changes were statistically non-significant | Moderate: focus on coverage despite non-significant mortality trends. Discussion focused on positive improvements. | Moderate |
| 9 | Garchitorena, et al. (2017) | Moderate: lack of random allocation can lead to unmeasured confounding | Moderate: intervention areas could be selected due to readiness and feasibility | Low: Intervention Cleary defined and implemented | Low: No major deviations reported | Moderate: Use of mixed population and system data can lead to inconsistencies, incomplete records, or recall issues | Moderate: data quality depends on reporting systems and population survey which can be inaccurate | Moderate: focused on positive improvements. Broader or null received less emphasis (maternity gains were smaller) | Moderate |
