# Supplementary material for: Primary health care networks and impacts in low- and middle-income countries: a systematic review
Source: Health Policy Plan. 2026 Jan 16;41(3):471–91. doi: 10.1093/heapol/czag003 (PMC12972678; doi:10.1093/heapol/czag003)
Supplement: czag003_Supplementary_Data [file czag003_supplementary_data.zip › Supplementary file 4.docx]

**Mixed Methods Appraisal Tool (MMAT) Quality assessment**

| **MMAT** | | **Screening questions** | | **Qualitative** | | | | | **Quantitative nonrandomized** | | | | |
| --- | --- | --- | --- | --- | --- | --- | --- | --- | --- | --- | --- | --- | --- |
| **N** | **Study** | **S1: Clear research questions?** | **S2. Do the collected data allow to address the research questions?** | **1.1. Is the qualitative approach appropriate to answer the research question?** | **1.2. Are the qualitative data collection methods adequate to address the research question?** | **1.3. Are the findings adequately derived from the data?** | **1.4. Is the interpretation of results sufficiently substantiated by data?** | **1.5. Is there coherence between qualitative data sources, collection, analysis and interpretation?** | **3.1. Are the participants representative of the target population?** | **3.2. Are measurements appropriate regarding both the outcome and intervention (or exposure)?** | **3.3. Are there complete outcome data?** | **3.4. Are the confounders accounted for in the design and analysis?** | **3.5. During the study period, is the intervention administered (or exposure occurred) as intended?** |
| 1 | Bhatta, et al. (2020) | Yes, the study aims to utilizes the four domains of effective Networks of Care (NOC) as a lens to describe  the suite of interventions of a biosocial approach to maternal and neonatal health services in rural  Nepal | Yes, monitoring data, policy reviews, facility audits, training records, social audits, case reviews, and community feedback | Yes, the case study is adequate for describing the collaboration | Partial, data came from various sources (MoUs, audits, social audits, community meetings, death reviews), but methodological detail is limited. | Partial, the data analysis process is not described, but findings are reported as coming from the data (e.g. document reviews, audits, community feedback) | Partial, little presentation of quotes or systematically analysed qualitative evidence | Partial, program data, social audits, and policy reviews align with objectives, but the integration and analytic process is not clearly explained | Partial, the paper reports an ongoing programme that still aims to expand: By the end of 2019, OHW had supported building or renovation  of 388 birthing centers in rural areas throughout Nepal, with plans to upgrade 500 additional facilities  to provide quality maternal and neonatal health services by 2030. Since 2010, the network has served  200,000 underserved and vulnerable pregnant women in the project catchment areas, including  52,000 in 2019 alone. It is anticipated that the program will support 71,000 deliveries in 2020. As the  project expands geographically to more populous regions of the country, the aim is to reach 230,000  pregnant women per year by 2030, or about onethird of the 700,000 births in Nepal annually | Yes, referral data, electronic health records (EHR) system for maternal health records, costs | Yes, data are input at each health facility, and OHW verifies the data for completeness and other issues  as part of their monitoring and supervision. The OHW monitoring mentors report that the data quality has generally been impressive | No, there is no description of data analysis and confounders | Yes, OHW interventions were delivered as planned (six-year cycle, training, mentoring, exit strategies) |
| 2 | Cordier, et al (2020) | Yes, the study aims to explore the MoPH and PIVOT (a non-governmental organization) collaboration in Ifanadiana District, Madagascar, focusing on elements of the collaboration across the four Network of Care domains: (I) agreement and enabling environment, (II) operational standards, (III) quality, efficiency, and responsibility, (IV) learning and adaptation | Yes, program monitoring data, routine health system information, facility assessments, referral statistics, and qualitative insights | Yes, the case study is adequate for describing the collaboration | Partial, multiple sources used (e.g., memorandum of understanding, facility assessments, routine data, community feedback), but limited detail on how qualitative data (interviews, narratives) were collected | Partial, the data analysis process is not described, but findings are reported as coming from the data (e.g. interviews with women and trusted sources of maternal health information) | Partial, little presentation of quotes or systematically analysed qualitative evidence | Partial, findings are supported by the evidence, but qualitative evidence is not clearly presented (e.g., quotations form interviews) | Yes, at the district level and at all levels of the health system in  Ifanadiana District | Yes, more than 800 indicators and geo-located information on utilization, coverage, and population health. Also, health management information system (HMIS) that captures system inputs and utilization at all facilities in  the district. The HMIS includes epidemiologic tracking that varies in frequency depending on disease outbreaks | Yes: Since its inception, PIVOT  has invested in a data and analytics platform that allows for extensive monitoring and evaluation,  rigorous quantitative epidemiology and operations research, technological tool development, and qualitative inquiry | No, there is no description of data analysis and confounders | Yes, Implementation seems consistent with the planned collaboration and NOC framework |
| 3 | D’Mello, et al. (2020) | Yes, the study aims to use the four domains of the Networks of Care framework to document the wide-ranging efforts made to build and maintain the Comprehensive Community Based Rehabilitation (CCBRT) Network of Care in order to solve for specific challenges in maternal and neonatal health service delivery in the urban context of the Dar es Salaam region in Tanzania | Yes, programmatic documentation provided by CCBRT and interviews  with key stakeholders | Yes, the case study is adequate for describing CCBRT network of care | Partial, source of data and NOC framework (Agreement and Enabling  Environment, Operational Standards, Quality, Efficiency and Responsibility, and Learning and Adaptation) are given, but methodological detail (e.g., sampling of interviews, analytic approach) are not described | Partial, the data analysis process is not described, but findings are reported as coming for the data (e.g. programmatic documentation provided by CCBRT and interviews  with key stakeholders) | Partial, little presentation of quotes or systematically analysed qualitative evidence | Partial, the use of routine data, interviews, and audits align with the study’s aims, but the integration and analytic rigor are not clear | Yes, facilities included 22 government hospitals and catchment facilities operating across Dar es Salaam, which were part of CCBRT | Yes, facility records and audits: data on routine care in the antenatal clinic, labour and delivery, and postnatal care; these include total deliveries, live births, maternal and perinatal death, referrals, obstetric procedures and value for money | Partial, CCBRT supported quarterly quality improvement meetings where the facility data from the network were presented and discussed, demonstrating how to use the data collected and generating data-driven decision-making and improvement plans. However, despite improvements in the quality, data quality was poor and lacked standardised indicators | No, there is no description of data analysis and confounders | Yes, the Regional–CCBRT partnership implemented interventions across the continuum of care that included clinical training in basic and comprehensive emergency obstetric care, investments in infrastructure, and a rigorous maternal and perinatal death audit and follow-up program |
| 4 | Hyre, et al. (2019) | Yes, the study aims to describe the Expanding Maternal and Neonatal Survival (EMAS) program in Indonesia. | Yes, blinded medical records, service statistics, maternal and newborn death audits, case reviews, mentoring records, and external evaluations. | Yes, the case study is adequate for describing EMAS network of care | Partial, source of data is given, but methodological detail (e.g., sampling of interviews, analytic approach) are not described | Partial, the data analysis process is not described and link between raw data and reported findings not entirely clear | Partial, findings rely on aggregated outcomes and program reports rather than evidence presentation | Partial, multiple sources used, but it is unclear how the data was integrated in the analysis | Partial, EMAS covered +400 facilities in 6 provinces, but selection was purposive (high-burden provinces where 50% of all maternal deaths were occurring) not representative nationally | Yes, facility service statistics, mortality data, quality of care, management of maternal and newborn complications, improvement of referral efficiencies for maternal and newborn complications | No, outcomes and impact of EMAS approaches over the term of the program are described in other articles | No, there is no description of data analysis and confounders | Yes, the program was implemented per its theory of change with mentoring, data-driven quality improvement, and referral strengthening |
| 5 | Fasawe, et al (2020) | Yes, the case study clearly aims to describe the Network of Care (NOC) in Northern Nigeria and Lagos, highlighting how each developed to address specific local needs | Yes, routinely reported data, program operational manuals, standard operating procedures (SOPs), and interviews with NOC stakeholders to retrospectively document the  two NOC according to the NOC definition and framework | Yes, qualitative case study is adequate to describe locally tailored NOC models in Northern Nigeria and Lagos | Partial, multiple sources such as interviews and review of institutional documents. However, methods are not described, and there is limited detail about interview design, participant selection, or data capture | Partial, the data analysis process is not described. But the authors report the findings from review of documents (e.g., Standard Operating Procedures) and interviews across some NOC domains | Partial, the link between interpretation and underlying data (interviews, field reports) is underdeveloped with very few verbatim quotes, for example | Partial, multiple sources are used, but the integration process is not clear, leading to uncertainty about the coherence between qual data sources, collection, analysis and interpretation | NA | NA | NA | NA | NA |
| 6 | Vergara, et al (2020) | Yes, the study clearly aims to explore the public-private Network of Care, Quirino Recognized Partners (QRP), and how QRP was established to solve particular contextual problems (e.g., congestion and poor maternal and neonatal health outcomes), with express attention to how actions taken related to the domains of Networks of Care | Yes, semi-structured interviews with relevant health care and other workers, institutional documents, observations, and field notes | Yes, a qualitative case study is appropriate for describing processes of partnership-building, trust, and implementation of a network of care | Partial, multiple sources such as interviews, review of institutional documents, observations, and field notes. However, methods are not completely described, and there is limited detail about interview design, participant selection, or data capture | Partial, the data analysis process is not described. While the report of the findings from interviews (e.g., President of the Philippines Obstetric and Gynaecology Society, midwives, obstetricians); review of documents (e.g., memorandum of understanding, Standard Operating Procedures, Operational Manual for QRP ) are clear, findings form observations and field notes are not clearly reported | Partial, the lack of clarity regarding the findings from observations and field notes makes it difficult to conclude that the results are supported by the data | Partial, data sources are consistent with the objectives, but the analytic process is not clearly explained (e.g., how themes were generated, whether coding framework was used). This limits assessment of methodological coherence | NA | NA | NA | NA | NA |
|  |  | *Score: It is discouraged to calculate an overall score from the ratings of each criterion. Instead, it is advised to provide a more detailed presentation of the ratings of each criterion to better inform the quality of the included studies. This may lead to perform a sensitivity analysis (i.e., to consider the quality of studies by contrasting their results). Excluding studies with low methodological quality is usually discouraged | | | | | | | | | | | |
